# The pivotal role of TMPRSS2 in coronavirus disease 2019 and prostate cancer

**DOI:** 10.2217/fon-2020-0571

**Published:** 2020-07-13

**Authors:** Veronica Mollica, Alessandro Rizzo, Francesco Massari

**Affiliations:** ^1^Division of Oncology, S.Orsola-Malpighi Hospital, Bologna, Italy

**Keywords:** COVID-19, prostate cancer, SARS-CoV-2, TMPRSS2

The coronavirus disease 2019 (COVID-19) pandemic represents a public health emergency of international proportions, which is having catastrophic consequences throughout the world [1]. To date, around 6 million confirmed cases have been diagnosed worldwide and over 370,000 people perished because of COVID-19 [2]. The clinical disease called COVID-19 is caused by the severe acute respiratory syndrome coronavirus 2 (SARS-CoV-2), a novel betacoronavirus sharing 79% sequence identity with SARS-CoV, the agent which provoked the 2003 SARS outbreak [1].

**Figure 1.**
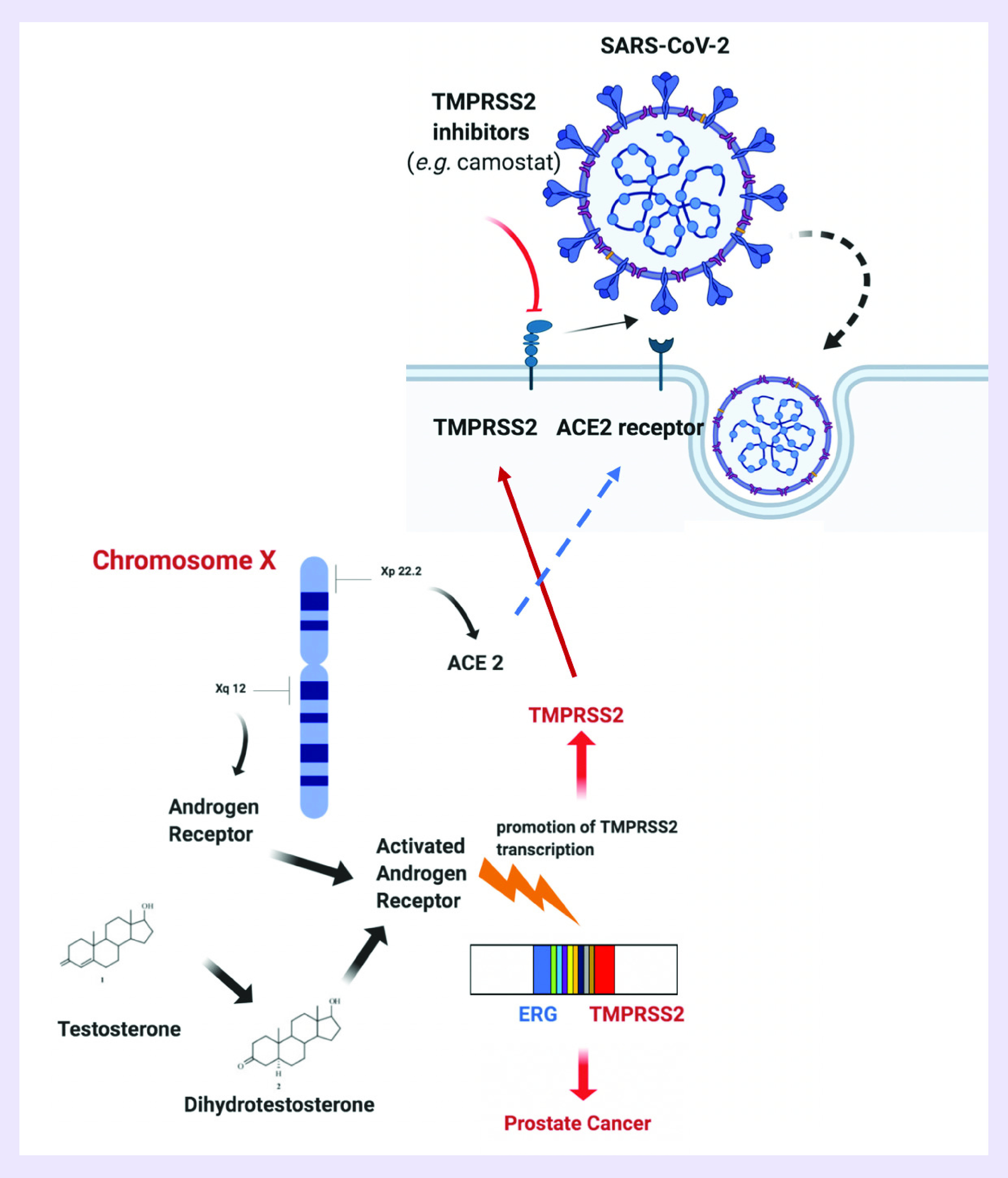
Regulation of *TMPRSS2* gene transcription and process of severe acute respiratory syndrome coronavirus 2 entry into target cells. Androgen receptor gene and *ACE2* gene are located on Xq12 and Xp22.2, respectively. Testosterone and dihydrotestosterone stimulate androgen receptor activity. Activated androgen receptor regulates transcription of *TMPRSS2* gene. *TMPRSS2:ERG* gene fusion is associated with prostate cancer development. SARS-CoV-2 engages ACE2 as the entry receptor and uses TMPRSS2 for spike protein priming. Serine protease inhibitors, like camostat mesylate, can inhibit TMPRSS2 and partially block SARS-CoV-2 spike protein-driven entry. SARS-CoV-2: Severe acute respiratory syndrome coronavirus 2.

Recent evidence suggested that SARS-CoV-2 uses the ACE2 receptor for cell entry, in synergy with the host’s TMPRSS2 [1]. More specifically, the viral S glycoprotein is cleaved by TMPRSS2, thus facilitating viral activation and representing one of the essential host factors for SARS-CoV-2 pathogenicity (Figure 1) [3]. This process is similar to viral activation and cell entry of other coronaviruses, including SARS-CoV, as well as influenza virus such as influenza H1N1 [4]. Despite several studies reported that *in vitro* activation of viral spikes could be mediated by other proteases such as TMPRSS4, TMPRSS11A, TMPRSS11D and TMPRSS11E1, TMPRSS2 activity is currently considered the sole crucial for cell entry and viral pathogenesis [3].

From the early days of coronavirus outbreak in China, significant differences in terms of severe cases and deaths have been reported between adult males and females (around 58 and 42%, respectively) [1]. Moreover, this pattern seems to be largely repeating itself, albeit with slightly different proportions, as indicated by recent epidemiological data across distinct continents and countries [4]. The international scientific community is investigating the reasons behind these gender differences, with biological and behavioral features (e.g., cigarette smoking) which have been called into question, including TMPRSS2 [3].

Interestingly, *TMPRSS2* gene has a pivotal role also in prostate cancer development and progression. TMPRSS2 is expressed on the luminal side of the normal prostatic epithelium and it is increased in malignant prostatic tissue. In particular, the *TMPRSS2*:v-ets avian erythroblastosis virus E26 oncogene homolog (*ERG*) gene fusion is present in about 50% of prostate tumors in Caucasian men and is frequently an early event in prostate carcinogenesis [5,6]. *TMPRS22* and *ERG* genes are located tandemly on chromosome 21q22 and expression of *TMPRSS2* is androgen-regulated and prostate-specific [7]. The fusion results from intrachromosomal deletion of ~3-Mb of the interstitial region between the *TMPRSS2* and *ERG* loci or from an insertion of the interstitial region causing a chromosomal rearrangement [7]. The role of this gene fusion is crucial in the development of prostate cancer because it leads to the activation of ERG transcription factor. In fact, in the presence of this gene fusion androgen-responsive elements within the *TMPRSS2* promoter drive the overexpression of *ERG*. *ERG* is a member of the E-twenty-six family members (*ETS*), master transcription factors that have a key role in differentiation, apoptosis, cell proliferation and inflammation. *TMPRSS2:ERG* fusion can cause cancer progression by disrupting the androgen receptor (AR) lineage-specific differentiation program of the prostate and favoring EZH2-mediated cellular de-differentiation [8]. The gene fusion can produce a repression of the AR signaling, thus creating a selective pressure that leads to the development of recurrent tumors with AR amplification and resistance to hormone deprivation therapies. *ERG* can directly induce epigenetic silencing of developmental regulators and tumor suppressor genes via direct activation of the polycomb group protein EZH2, thus favoring EZH2-mediated stem cell-like de-differentiation [8]. Moreover, this gene fusion often coexists with phosphatase tensin homolog (*PTEN*) loss, thus promoting the development of invasive carcinoma [5]. *TMPRSS2:ERG* fusion positive prostate tumors have also been associated with activation of the NOTCH pathway that promotes proliferation and maintenance of progenitor cells in the developing prostate. This finding could have therapeutic implication since NOTCH1 inhibition can reduce cell growth and invasion [5]. The prognostic and predictive role of *TMPRSS2:ERG* fusion has been widely investigated. The presence of this fusion has been associated with poor survival outcomes, aggressive disease and biochemical recurrence [9]. Furthermore, *TMPRSS2:ERG* fusion was not found to be predictive of response to abiraterone acetate in metastatic castration resistant prostate cancer patients [10].

It is recognized that SARS-CoV-2 infection, similarly to SARS-CoV, is transmitted through respiratory droplets which penetrate in the upper respiratory tract [11]. As stated above, TMPRSS2 seems to play a crucial role in SARS-CoV and SARS-CoV-2 pathogenesis; moreover, despite TMPRSS2 expression is several times higher in prostate epithelium compared with any other tissue, TMPRSS2 has been found also in the aerodigestive tract [12]. Interestingly, differences in TMPRSS2 expression in lung cells may vary across different populations, an element which could be implied in susceptibility to coronavirus and influenza virus infections. Thus, revealing how the expression of TMPRSS2 protein in the lung could vary between men and women has been indicated as an important element in understanding differential susceptibility to SARS-CoV-2 infection. Nonetheless, several studies have reported controversial results, most of them suggesting that constitutive expression of TMPRSS2 in lung cells do not seem to differ according to gender [13,14].

Here, we investigate ACE2 and TMPRSS2 expression levels and their distribution across cell types in lung tissue (12 donors, 39,778 cells) and in cells derived from subsegmental bronchi al branches (four donors, 17,521 cells) by single nuclei and single cell RNA sequencing, respectively. While TMPRSS2 is strongly expressed in both tissues, in the subsegmental bronchial branches ACE2 is predominantly expressed in a transient secretory cell type. Interestingly, these transiently differentiating cells show an enrichment for pathways related to RHO GTPase function and viral processes suggesting increased vulnerability for SARS-CoV-2 infection. Our data provide a rich resource for future investigations of COVID-19 infection and pathogenesis

A report by Bertram *et al.* suggested that TMPRSS2 is less expressed in Type II alveolar cells and alveolar macrophages than in bronchial epithelial cells [15]; moreover, this study found no expression of TMPRSS2 protein in Type I alveolar cells of the respiratory surface. In this context, another key element to consider could lie in the modifications of lung TMPRSS2 expression caused by viral infections, as suggested by previous findings on SARS-CoV and ACE2 receptor [16]. Moreover, single nucleotide polymorphisms (SNPs) have been linked to higher expression of TMPRSS2 protein, something which has been associated with an increased susceptibility to influenza virus infection [17].

A recent study by Song *et al.* analyzed gene co-expression of ACE2 and TMPRSS2 in 24,519 human prostate cells, finding that the 0.61% of club cells and the 0.40% of hillock cells co-expressed TMPRSS2 and ACE2 [18]. The authors conducted the same analysis in human lung club cells, human lung secretory cells and murine lung club cells; the report found that higher TMPRSS2 and ACE2 co-expression was detected in males pneumocytes I/II compared with female cells, representing an interesting evidence which could play a role in gender ‘pattern’ of COVID-19.

Another recent study by Lukassen *et al.* investigated ACE2 and TMPRSS2 expression levels across 39,778 lung cells and in 17,521 cells derived from subsegmental bronchial branches [19]. Although TMPRSS2 expression resulted high in both tissues, subsegmental bronchial branches cells showed higher ACE2 expression, especially in transient secretory cells; more specifically, transient secretory cell types presented an enrichment of RHO GTPases with their relative pathways. Most notably, RHO GTPases have been associated, according to previous studies, with membrane remodeling and viral cycle, especially in terms of entry, replication and viral spread [20,21]; thus, transient secretory pulmonary cells could be particularly vulnerable to SARS-CoV-2 infection.

Since COVID-19 is a disease previously unknown to human beings, no proven treatments exist at present and the most important public health solution would be an effective vaccine [1]. However, the male preference of SARS-CoV-2 and the androgen-dependent expression of TMPRSS2 indicate that targeting TMPRSS2 could emerge as a novel option to treat COVID-19, as in the case of SARS-CoV and influenza virus [4].

A recent study by Montopoli *et al.* analyzed data on 9280 SARS-CoV-2 positive patients, of which 4532 (44%) males and 118 (1.3%) with prostate cancer [22]. Males developed more severe complications, were more frequently hospitalized (men 60% and women 40%), and accounted for more deaths (men 62% and women 38%). Prostate cancer patients receiving androgen deprivation therapy (ADT) had a significantly lower risk of SARS-CoV-2 infection compared with patients who did not receive ADT (odds ratio: 4.05; 95% CI: 1.55–10.59). This study sets the scenario for further studies assessing the role of antiandrogens commonly used for prostate cancer patients to prevent or treat COVID-19.

Another recent study in 19 high-volume medical oncology departments in Italy examined the frequency of COVID-19 in prostate cancer patients undergoing ADT, either alone or in combination with another agent [23]. A concomitant confirmed diagnosis of SARS-CoV-2 infection was found in 1.8% (36 out of 1949 total patients) of the examined population. ADT did not seem to have a protective effect considering the higher lethality of SARS-CoV-2 in patients <70 years (25% in prostate cancer patients compared with 13% in infected Italian males). The findings of these two analyses on prostate cancer patients seem conflicting [22,23], supporting the need for further retrospective or prospective evidence on the role of antiandrogen agents in patients with COVID-19.

Expression levels and variants in *ACE2* and *TMPRSS2* genes have been investigated for their possible role in affecting COVID-19 severity in a large Italian cohort [24]. Italians resulted to have a significant decrease in the burden of deleterious variants compared with Europeans. One the four SNPs with significant different frequency in Italians compared with East Asians and Europeans is the missense substitution p.Val160Met, that is associated with genomic rearrangements involving *TMPRSS2* associated with higher risk of prostate cancer [25]. Moreover, in the analysis by Asselta *et al.* two haplotypes (the first composed at least of SNPs rs463727, rs34624090, rs55964536, rs734056, rs4290734, rs34783969, rs11702475, rs35899679 and rs35041537; the second composed of three SNPs, rs2070788, rs9974589 and rs7364083) associated with upregulation of *TMPRSS2* gene expression resulted to be more frequent in Italians than in the East Asian population [24].

These findings are of particular interest considering the putative role of *TMPRSS2* in SARS-CoV-2 infection. Considering that the expression of *TMPRSS2* gene is modulated by estrogens and androgens, hormone receptors signaling antagonists could be explored as treatments strategies against COVID-19 for their role in downregulating *TMPRSS2*. In this line, men with prostate cancer presenting *TMPRSS2:ERG* fusion and receiving androgens receptor signaling inhibitors could represent a subset of patients with reduced risk of SARS-CoV-2 infection or severity [26].

Regarding treatment implications, further data are needed to assess androgen targeting agents in the management of COVID-19. Evaluating *TMPRSS2* gene expression in lung tissue in patients with prostate cancer undergoing antiandrogen agents could provide preliminary data to elaborate clinical trials assessing these agents in COVID-19 patients.

In a recent *in vitro* study by Hoffmann *et al.*, the TMPRSS2 inhibitor camostat mesylate blocked the SARS-CoV-2 entry into primary lung cells, suggesting that TMPRSS2 could represent a potential target in SARS-CoV-2 treatment [3]. Nonetheless, previous studies have suggested no gender differences in TMPRSS2 mRNA expression levels in pulmonary tissue [4]; moreover, some authors reported that normal female androgen levels could be able to regulate TMPRSS2 transcription, and similarly, TMPRSS2 also seems to respond to estrogenic stimulation [3].

Since serine proteases inhibitors such as camostat mesylate and nafamostat are able to block TMPRSS2, these antiandrogens could help in developing novel measures to inhibit viral entry and pathogenesis in an *in vivo* setting [4]. Studies are required with the aim to investigate the association between the severity of COVID-19 disease and androgenic activity, a connection which could represent the source of novel potential treatments for COVID-19 infection.
